# Examining the relationship between diet-induced acidosis and cancer

**DOI:** 10.1186/1743-7075-9-72

**Published:** 2012-08-01

**Authors:** Ian Forrest Robey

**Affiliations:** 1 Arizona Respiratory Center, University of Arizona, 1501 N. Campbell Ave., Suite 2349, PO Box 245030, Tucson, Arizona 85724, USA

**Keywords:** Acid-base balance, Diet, Acidosis, Cancer

## Abstract

Increased cancer risk is associated with select dietary factors. Dietary lifestyles can alter systemic acid-base balance over time. Acidogenic diets, which are typically high in animal protein and salt and low in fruits and vegetables, can lead to a sub-clinical or low-grade state of metabolic acidosis. The relationship between diet and cancer risk prompts questions about the role of acidosis in the initiation and progression of cancer. Cancer is triggered by genetic and epigenetic perturbations in the normal cell, but it has become clear that microenvironmental and systemic factors exert modifying effects on cancer cell development. While there are no studies showing a direct link between diet-induced acidosis and cancer, acid-base disequilibrium has been shown to modulate molecular activity including adrenal glucocorticoid, insulin growth factor (IGF-1), and adipocyte cytokine signaling, dysregulated cellular metabolism, and osteoclast activation, which may serve as intermediary or downstream effectors of carcinogenesis or tumor promotion. In short, diet-induced acidosis may influence molecular activities at the cellular level that promote carcinogenesis or tumor progression. This review defines the relationship between dietary lifestyle and acid-base balance and discusses the potential consequences of diet-induced acidosis and cancer occurrence or progression.

## Diet, cancer, and ‘acidity’

The relationship between diet and cancer is well known [1-3]. Dietary intake exists as the largest external or environmental epigenetic factor capable of driving the development or maintenance of cancer. The American Institute for Cancer Research (AICR) comprehensive global report has compiled numerous studies demonstrating associations between dietary habits and cancer risk [4]. The findings recommend increased or regular consumption of vegetables, fruits, whole grains, and legumes, while discouraging excess consumption of sugary and energy-dense foods and drinks, red and processed meats, and salty processed foods (http://www.aicr.org).

Acidity is a well known factor associated with cancer. Lower pH levels in the extracellular space promote the invasive and metastatic potential of cancer cells [5-14]. Extracellular acidity is mostly generated by tumor cells due to upregulated proton [H^+^ and lactic acid production [15]. This phenomenon is distinct from ‘acidity’ caused by a net-acid diet. A net-acid diet or acidogenic diet is determined by the balance between acid and base-forming dietary constituents. Most fruits and vegetables are net-base producing foods since the metabolized products are organic anion precursors such as citrate, succinate, and conjugate bases of carboxylic acids [16-18]. The final metabolite of these precursors is bicarbonate anion. Sulfur containing amino acids, methionine and cysteine, typically found in meats, eggs and dairy products, are oxidized into sulfuric acid which is ultimately net-acid producing [16]. Cationic amino acids such as lysine and arginine can be acid producing if their anionic counterpart is chloride, sulfate, or phosphate. However, if the anionic component is a metabolizable organic acid (glutamate or aspartate), there is almost no impact on systemic acidity [17,18]. Other dietary factors are known to influence acid-base status as well. Sodium chloride is reported to be an independent and causal factor for inducing metabolic acidosis in a dose-dependent manner [19,20]. Conversely, potassium salts, and to a lesser degree magnesium, serve as a countervailing effect on net acid excretion and help to promote alkaline balance [21,22].

Acidogenic dietary intake such as high protein consumption can have an immediate effect on increasing net acid production while low protein lacto-vegetarian consumption can result in significantly reduced net acid excretion [23,24]. Short-term dietetic acid loading may cause temporary acid-base disequilibrium, but is quickly compensated and has no measureable clinical effect. A persistent acidogenic diet, however, raises the likelihood of an increased [H^+^ surplus and chronically lower levels of serum bicarbonate if compensatory processes become less efficient and are unresolved by dietary adjustments. Potential long-term effects of acidogenic diets are further compounded by the reduction of renal function typically from ageing [16,25-28].

Blood pH from prolonged or chronic acidogenic diets is reported to be near the lower physiological range (7.36-7.38) rather than the higher end (7.42-7.44). Specifically, persistent acidogenic diets have the potential to cause small decreases in blood pH and plasma bicarbonate, but not beyond the normal physiological range. This condition is described as ‘diet-induced’, ‘low-grade’, or ‘chronic metabolic acidosis’ [28-30] or sometimes ‘latent acidosis’ [31]. Diet-induced acidosis is distinct from clinical metabolic acidosis in that clinical metabolic acidosis occurs when factors other than just acidogenic diet contribute a system’s inability to compensate for blood [H^+^ perturbations, typically resulting in blood pH below 7.35 [32]. The patho-physiological effects of clinical metabolic acidosis are well known [33], while the true pathophysiological impact of long-term, diet-induced acidosis is not well understood. For example, it is unknown if [H^+^ accumulation from chronic diet-induced acidosis can be stored at the cellular level if it does not play a role in lowering blood pH or is compensated by competent renal or respiratory function. Studies of the impact of clinical metabolic acidosis on biological systems may still be informative towards understanding the effects of diet-induced acidosis because they examine how acid-base disequilibrium causes physiological stress and influences molecular pathways active in disease processes [34].

It is generally understood that the cancer condition evolves from genetic and epigenetic changes in the normal cell. Both microenvironmental and systemic factors exert selective pressures that aid in the initiation or aggravation of tumors. Acid-base disequilibrium is considered a type of systemic stress. With the understanding that long-term acidogenic diets potentially exert chronic physiological stress, the question proposed here is: Can diet-induced acidosis increase cancer risk or promote existing tumors?

## Cortisol

### Cortisol and acid-base balance

Acid-base balance in the body influences adrenal hormone production of cortisol. When bicarbonate [HCO_3_^-^ levels are low the kidneys upregulate glutaminase activity and trigger cortisol production [35-37]. Studies in animals and humans have reported that system cortisol levels are enhanced by acid-base disruption through transiently induced metabolic acidosis [37]. Acidosis appears to mediate cortisol activity through the pituitary-adrenal cortex-renal glutaminase I axis [37]. Dietary induction of acidosis increases serum cortisol concentrations [38]. In healthy adult humans serum and salivary cortisol is increased significantly within hours after a high protein meal, and cortisol levels were dependent on the protein content of the meals [39,40].

The converse to these findings is shown in a study designed to neutralize the acidogenic effect of the ‘Western’ diet, characterized by a high consumption of meat, salt, sugar and fat, and proportionately lower intake of fruit, vegetables, and whole grains. The relationship to cortisol levels and acid-base status were examined in six healthy men and three women measuring serum and urine cortisol concentrations along with cortisol metabolite levels (tetrahydrocortisone and tetrahydrocortisol) in the urine of individuals with sodium and potassium chloride replaced with equimolar amounts of sodium and potassium bicarbonate in an otherwise similar diet under “metabolic ward conditions”. Within 24 hours, urinary and plasma cortisol and corresponding metabolites were significantly lower, signaling lower cortisol production and activity. Urinary pH and serum [NaHCO_3_^-^ levels increased while serum pH remained stable [41].

Not all studies report a positive correlation between high protein, potentially acidogenic diets, and cortisol levels. These studies did not assess acid-base balance in their experimental populations so it is difficult to confirm if these studies are directly comparable to findings linking acidogenic dietary intake and increased cortisol production. It is likely that factors such as gender and body mass index are relevant inconsistencies between various reports [42-44].

Many of these studies suggest there may be a role for diet-induced acidosis in modulating systemic cortisol levels, and that neutralization of acid loading through alkalinization may reduce cortisol levels. Moreover, most of the studies evaluating the role of acidogenic intake on cortisol demonstrate that the interventions had an acute and dose-dependent effect on cortisol levels, suggesting a direct or closely linked dynamic between acid-base status and system cortisol levels. Finally, the studies show that diet-induced acidosis is mild and subsequent induction of cortisol activity, although higher in serum concentration, is sub-clinical and within the normal serum range [41]. If there were pathophysiological consequences it could only be derived from chronic or persistent conditions maintained on an acidogenic diet.

### Cortisol bioactivity in cancer

There is no clear mechanism linking cortisol bioactivity directly with carcinogenesis, but studies have reported that cortisol signaling may exert biological influence on existing tumors. Androgen-independent prostate tumors expressing high levels of androgen receptor can be stimulated by cortisol and its metabolite cortisone, resulting in growth promotion and proliferation. The mechanism for this interaction is made possible due to a mutation in the androgen receptor that favors ‘promiscuous’ binding of additional signaling molecules like glucocorticoids [45]. In some breast cancer studies glucocorticoids suppress growth by blocking cell cycle progression [46]. Tumor inhibition appears to be androgen dependent at least in some cell lines [47,48]. In colon cancer, cortisol signaling inhibits 11β-hydroxysteroid dehydrogenase 2 (11βHSD2) enzymatic activity which prevents activation of the COX-2 tumor promoter, an early activation marker for colon carcinogenesis. The diversity of tumor responses to glucocorticoid signaling suggests that the relationship between hormonal activity and tumor regulation is receiver-dependent. The following sub-sections discuss the possible indirect role of cortisol signaling in cancer risk and carcinogenesis.

### Cortisol and tryptophan metabolism

Cortisol activates the tryptophan metabolism pathway which is carried out by rate-limiting enzymes of tryptophan catabolism, 2,3-dioxygenase (TDO) and indoleamine 2,3-dioxygenase (IDO). Cortisol directly stimulates TDO activation and may augment IDO activity indirectly through inflammatory cytokine signaling such as interferon gamma [49,50]. Excessive or chronic cortisol production acquired from a ‘Western’ dietary lifestyle could play a role in augmenting the tryptophan metabolism pathway and drive downstream molecular events that promote carcinogenesis.

The product of TDO and IDO activity, kynurenine, has several roles in promoting tumorigenesis. Kynurenine inhibits the activation of effector T-cells when tryptophan levels are low. Incapacitating effector T-cell function is suggested as an important component in increasing vulnerability to tumor development [51-53]. Tryptophan metabolism also promotes immune tolerance of professional antigen presenting cells (APCs) which are critical in activating other immune cells [51,53,54]. Finally, kynurenine binds to aryl hydrocarbon receptor (AHR), which mediates TDO and IDO signaling in regulatory T-cells. The activated AHR suppresses the stimulation of regulatory T-cells involved in inhibiting early tumor development [51,55-57]. The connection between diet-induced, low-grade hypercorticoidism and the effect on tryptophan metabolism to subsequently promote tumor development has not been adequately explored. Furthermore, it is unknown what other factors may enhance, regulate, or attenuate these signaling events, but a persistent reduction of effective immune surveillance capability promoted indirectly by diet-induced acidosis could cultivate microenvironmental conditions favorable for oncogenic cells to develop metastatic potential.

### Cortisol and insulin resistance

Upregulated cortisol bioactivity driven by diet-induced acidosis may be a factor in metabolic syndrome by promoting insulin resistance. Chronic hyperglucocorticoidism upregulates visceral obesity while reducing insulin sensitivity mainly in visceral adipocytes which appear to be more responsive to cortisol than subcutaneous adipocytes due to higher expression levels of glucocorticoid receptors [58,59]. Visceral adipocytes also exhibit greater 11βHSD1 activity, which converts cortisone to bioactive cortisol [60]. Glucocorticoids stimulate visceral adipocytes to increase the activity of lipoprotein lipases, while simultaneously suppressing insulin mediated glucose uptake [61-66]. This phenomenon suggests that cortisol activated adipocytes are less efficient in storing fatty acids which tend to increase the level of free fatty acids in circulation and contributes to diminished insulin sensitivity [67].

Glucocorticoid signaling promotes insulin resistance through other signaling pathways as well. Insulin stimulated glucose transporter-4 (GLUT-4) translocation to the cell surface of adipose tissue is suppressed by glucocorticoids. Cortisol directly inhibits insulin secretion from pancreatic beta cells. Finally, cortisol can reduce insulin mediated vasodilation of endothelial cells, and suppresses peripheral insulin driven glucose uptake [68-70].

Acidosis associated insulin resistance through cortisol activity may result in compensatory pancreatic insulin secretion and higher levels of circulating insulin in the serum, a condition known as hyperinsulinemia. Epidemiology studies have shown a positive correlation between circulating insulin levels and increased risk and pathogenesis of colorectal and pancreatic cancers [71-76], cancers of the endometrium [77], kidney cancer [78] and breast cancer [79,80]. Longitudinal studies report a higher risk for breast cancer in women with hyperinsulinemia [81-83]. Human studies are confirmed by experimental data showing that injected insulin promotes tumorigenesis in animal models for colon [84] and breast [85,86] cancer. Insulin deficiency or insulin blocking reduces tumor incidence or progression and is reversible with re-introduction of insulin [87]. Several of the study findings conclude that hyperinsulinemia is an independent risk factor from obesity and diabetes [88].

Insulin is a pleiotropic hormone with both mitogenic and metabolic properties. It binds with the highest affinity to its own receptor and with lower affinity to the insulin growth factor-1 (IGF-1) receptor. The insulin receptor exists in two isoforms, IR-A and IR-B. IR-A is expressed at lower levels than IR-B, but has greater mitogenic activity when stimulated by insulin. Additionally, both insulin receptor isoforms can form heterodimeric complexes with the IGF-1 receptor. The IR-A/IGF hybrid receptor is expressed in all human tissues and binds to insulin with high affinity [89]. Activation of these receptors by insulin stimulates cellular transformation and promotes malignancy. Insulin promotes cellular proliferation, migration, and cellular survival mainly through the MAPK pathway and sometimes through PI3K pathway [88]. It is proposed that chronically exposed cells to even moderately elevated insulin levels may favor cell proliferation and subsequently increase the risk for malignant transformation [89]. Thus, persistent diet-induced acidosis favorable for maintaining chronically high levels of cortisol could be supportive of insulin sensitized tumor development.

### Insulin growth factor

Studies examining the relationship between diet-induced acidosis and insulin growth factor (IGF-1) levels have varied outcomes. Acute induction of systemic acidosis appears to reduce serum IGF-1 levels. Short-term (5-7 days) induction of metabolic acidosis in healthy male subjects using ammonium chloride (NH_4_Cl) causes a significant reduction in serum IGF-1 levels [90], confirming the results of an animal study carried out under similar parameters [91]. An adult fasting between 5-10 days induces a mild metabolic acidosis and appears to have the effect of reducing plasma IGF-1 concentrations as well [92-94]. Plasma IGF-1 levels are doubled by treatment with bicarbonate in individuals with renal tubular acidosis [95]. In healthy subjects though, neutralization of diet-induced acidosis with bicarbonate treatment for a 7 day period does not have a significant impact on IGF-1 levels [41].

High protein consumption over long-term periods (months to years), which promotes greater net acid production and subsequent latent or low-grade metabolic acidosis, appears to have the opposite outcome from short term studies on IGF-1 levels. Studies conducted for 12 weeks or longer revealed a strong correlation between increased dietary protein and higher serum IGF-1 levels, suggesting at least long-term dietary habits, not short-term perturbations, significantly impact IGF-1 serum concentrations [96-99]. Another epidemiological study in healthy middle-aged and elderly male participants concluded that while protein consumption was positively correlated to serum IGF-1 levels, the finding was only consistent in individuals with a body mass index (BMI) of <25kg/m^2^. There was no significant relationship between protein consumption and IGF-1 levels in obese individuals (BMI >25kg/m^2^). The study also reported that even while protein consumption increased IGF-1 serum levels, there was an age dependent decline in IGF-1 levels overall [100]. The findings suggest a potential for chronic acidogenic or ‘Western’ diets to elevate IGF-1, but other factors complicate this dynamic and require additional study. While it is reasonable to predict that the individuals in these long-term studies have developed low-grade acidosis from their diet, it does not mean that acidosis is a driver of IGF-1 upregulation. Furthermore, if diet-induced acidity upregulates IGF-1, as suggested from the long-term dietary studies, it is not yet determined if this occurs directly or indirectly through cortisol signaling [2,88,89].

IGF-1 binding to the insulin receptor has been shown to inhibit apoptosis and increase target cell proliferation, thus linking its signaling activity to the risk of different forms of cancer [101-103]. Several case control studies have demonstrated a possible link between IGF-1 bioactivity and different cancers including prostate [104,105], colorectal [106-108], and breast [109]. The serum IGF-1concentrations in the case population of the studies were relatively consistent with the ranges measured in the previously discussed studies evaluating the effects of ‘Western’ diet consumption on IGF-1 levels [97,100]. IGF-1 median levels of about 200ng/ml in individuals younger than 70 years of age were typically associated with high protein diets (~90-105 g/day).

## Adipokines

### Leptin

Leptin is an adipocyte derived hormone cytokine that plays a role in regulating body weight and energy balance in the hypothalamus [110]. Metabolic acidosis modulates lipid metabolism in adipocytes [111-114]. Acidosis reduces leptin concentrations in cultured adipocytes [112]. In uraemic Wistar rats, sodium bicarbonate supplementation appeared to increase (but not significantly) leptin levels [113]. A study in chronic kidney disease (CKD) patients with metabolic acidosis revealed that serum leptin was significantly increased by treating patients with a daily low to moderate dose (0.05-0.2g/kg) of sodium bicarbonate. The report concluded that either reversal of acidosis increases serum leptin or metabolic acidosis masks serum leptin levels [114].

Studies comparing serum leptin between healthy individuals consuming acidogenic type diets and those consuming more alkaline types of diets present mixed and variable findings. A study in men and women compared serum leptin levels between a group of 279 people consuming a diet rich in fish and a group of 329 people consuming a strictly vegetarian diet. Fish consumption is a high net acid producing diet [16]. Both groups had similar BMI values. The study was consistent with findings from *in vitro*, and animal investigations in that it reported the protein-rich diet was associated with significantly lower levels of serum leptin than in individuals on the vegetarian diet, independent of age [115]. However, another study measuring serum leptin levels in over 50,000 healthy participants reported a positive correlation with consuming a ‘Western’ diet, at least in the 5^th^ quintile of the study population [116]. Other studies have not observed an independent association between dietary intake and serum leptin levels after adjusting for energy intake, gender, age and BMI [117,118]. These reports illustrate the deep and complex relationship between acidogenic diets and serum leptin concentrations in humans.

Physiological acidosis may indirectly influence leptin activity through cortisol signaling in obesity which is a condition predicted to be associated with dysregulated acid-base balance [34]. As discussed previously, acid-base status affects cortisol levels [41]. In turn, cortisol stimulates synthesis and secretion of leptin directly from adipocytes [119]. Plasma leptin concentrations are positively correlated to body fat mass in humans [120,121]. Leptin has been shown to negatively regulate cortisol levels in healthy mice and humans [122-125], implicating leptin as an anti-obesity factor. In humans, leptin attenuation of cortisol appears to be a greater factor in females [124]. Serum leptin levels are paradoxically high, however, in obese individuals. This phenomenon is likely due to an acquired leptin signaling resistance that eventually occurs in the obese state [126]. On average, plasma leptin concentrations are 10 times higher in obese individuals compared to those of lean individuals [127,128].

Elevated plasma leptin levels in obesity may contribute to cancer incidence [129]. Leptin has been implicated as a functional component of mammary carcinoma in wild-type p53 deficient mice [130]. Epidemiological, animal, and *in vitro* studies have demonstrated that leptin is associated with breast cancer, prostate cancer, gynecological cancers, gastrointestinal cancers, and leukemia [131-133]. Leptin has numerous molecular targets allowing for a multifunctional effect. Leptin functions as a mitogen and is known to stimulate breast tumor cells, prostate tumor cell lines, as well as colonic and hepatic cells. Leptin signaling is most likely to activate the mitogen-activated protein kinase (MAPK) pathway through binding of Ob-Rb leptin receptor [131-138]. Leptin may also enhance cell proliferation through protein kinase C alpha (PKC-α) [139,140]. Leptin has been shown to bind the estrogen receptor and stimulate estrogen biosynthesis by induction of aromatase activity [141,142]. Other cancer-permissive functional activities of leptin include promotion of angiogenesis [143-145], apoptosis [146], and cellular migration [147].

### Adiponectin

Acid-base balance may play a role in modulating serum levels of adipokine hormone adiponectin. Adiponectin regulates multiple metabolic processes and is expressed exclusively in mature adipocytes and circulates in the plasma [148]. Numerous human and animal studies have reported a strong correlation between diet and serum adiponectin levels. Higher levels of serum adiponectin are typically associated with the ‘Mediterranean’ diet, known for high vegetable and fruit intake and low or moderate amounts of meat consumption. Other nutritional factors such as the amount and type of fatty acid intake are thought to influence serum adiponectin, but the mechanisms of diet-induced regulation of adiponectin regulation are not fully understood [149]. The first and only study demonstrating the role of acid-base disequilibrium in regulating serum adiponectin concentrations was an interventional trial to measure levels of serum adiponectin in healthy individuals induced with transient metabolic acidosis. Twenty healthy females completed a seven day course of oral ammonium chloride (NH_4_Cl), resulting in reduced serum bicarbonate and subsequent reduction in adiponectin mRNA and serum protein adiponectin. This was further confirmed in cultured adipocytes where acidosis inhibited gene transcription of adiponectin, suggesting a pH sensing mechanism at the cellular level may influence the regulation of adiponectin production [111].

Low serum adiponectin levels are considered to be permissive for development of cancer [3,150]. Reduced serum adiponectin levels are observed in patients with breast and gastric cancers, and simultaneously linked to dietary lifestyle [151,152]. Higher serum adiponectin may be protective against cancer as an anti-proliferative through direct binding of other growth factors, such as platelet derived growth factor-BB (PDGF-BB), heparin-binding epidermal growth factor-like growth factor (HB-EGF), and basic fibroblast growth factor (basic FGF), hence restricting bioavailability [153]. This was demonstrated in a mouse study where adiponectin was shown to slow tumor growth through its inhibitory effect on tumor neovascularization [154].

In addition to its interference with proliferative signaling, adiponectin mediates its regulatory effects through two receptors, AdipoR1 and AdipoR2 [155]. Signaling through these receptors stimulates the activity of adenosine monophosphate-activated protein (AMP-k) kinase and peroxisome proliferator-activated receptor alpha (PPARα) which drives glucose uptake and fatty acid oxidation. Through this mechanism, coupled with AdipoR1 receptor association with the insulin receptor, adiponectin is proposed to enhance signal transduction to promote insulin sensitivity [156]. Although a greater understanding is necessary, there is evidence suggesting acid-base status maintained through dietary intake could promote carcinogenesis or tumor progression through dysregulated adiponectin signaling.

### Lactic acid

A very recent discussion about the role of diet-induced acidosis and pathophysiology introduces the hypothesis that persistent acidogenic or ‘Western’ diets lead to latent or low-grade metabolic acidosis, subsequent acid-base balance disequilibrium, and production of lactic acid at the cellular level. These events appear to be critical upstream precursors to a host of ill-conditions, diseases, and ageing. The premise further explains that increased [H^+^ accumulates persistently in the mitochondrial matrix without contributing to ATP production. This dynamic is theorized to inhibit mitochondrial energy production (MEP) through inhibition of the TCA cycle. MEP inhibition results in the diversion of electrons away from completion of the electron transport chain and toward the reduction of oxygen (O_2_) into reactive oxygen species (ROS) such as free radical oxygen species or peroxides [34,157]. As this cycle continues, vulnerable cells develop a reduced capacity to restore homeostatic balance and are subject to increased intracellular oxidative stress.

The oxidative stress generated by ROS has multiple effects causing damage to cellular and organelle membranes, sulphydryl groups in proteins, and cross-linking or fragmenting ribonucleoproteins and DNA. DNA mutagenesis through persistent oxidative stress is generally accepted as a major mechanism behind carcinogenesis and cancer progression [158]. Oxidative DNA damage has been associated with breast cancer [159,160], hepatocellular carcinoma and liver cancer [161,162], and prostate cancer [163-165]. Oxidative stress in correlation with obesity can manifest and have significant pathogenic effects within the first two decades of life [166]. Although oxidative stress can be measured directly and indirectly through various methods, it is far more difficult to differentiate between acidogenic diet-induced and endogenous ROS production coupled with antioxidant status and other molecular factors that may impact oxidative steady state [167].

### Osteoclast activation

Although not fully understood, the long-term effect of diet-induced acidosis is considered to have an impact on bone osteoclasts [28]. Serum [HCO_3_^-^ concentrations may only partially account for neutralization of acidity, and may be supplemented further by alkaline stores from the soft tissue and bone [168]. Osteoclastic resorption of minerals is a proposed mechanism in buffering systemic acidosis [169,170]. *In vitro* findings demonstrating the mechanisms of excess [H^+^ on bone tissue is the most reliable evidence currently driving the concept of compensatory buffering through acidosis-induced bone resorption. In cultured osteoclasts, lower pH conditions induce the breaking up of mineralized bone tissue matrix [171-175]. Bicarbonate [HCO_3_^-^ deficiency may be sufficient to acidify media and promote net [H^+^ influx into bone [176], and appears to be necessary (not just reduced pH conditions which could be induced by respiratory acidosis) to stimulate calcium [Ca_2_^+^ efflux from bone [177].

Stimulation of osteoclastic resorption by diet-induced acidosis is mediated through receptor activator of NFкB ligand (RANKL) signaling [178]. RANKL signaling is known to promote osteoclast differentiation and activates various mitogenic pathways that are frequently operational in tumor cells, including p38, MAPK, AP-1, c-Jun, and Akt/PKB [179-182]. RANKL expression has also been observed in lymphoid tissue, skeletal muscle, thymus, liver, colon, intestine, heart, brain, and the adrenal and mammary glands [183]. RANKL signaling has been shown in mouse models to promote tumorigenesis in breast and lung tissue [184]. It is unknown, however, if systemic acidosis induces RANKL activity in other cell types besides osteoclasts.

One of the strongest RANK-stimulated transcription factors in osteoclasts is the nuclear factor of activated T-cells (NFATc1) protein [185,186]. Once exposed to extracellular acidic pH, cytosolic [Ca_2_^+^ stores in osteoclasts increase intracellular localization of nuclear transcription factor NFATc1 through calcineurin signaling. The ovarian cancer G-protein coupled proton-sensing receptor (OGR1), which is induced during osteoclast differentiation, is thought of as the primary mediator between acidosis and NFATc1 activation. Calcineurin signaling is not required however, to maintain NFATc1 activation under extracellular acidic conditions and NFATc1 activity is reversed by extracellular alkaline conditions, suggesting that acidosis directly prevents NFATc1 inactivation by kinases [187]. NFATc1 has many functions in cancer [188] and has been linked to regulation of the c-Myc oncogene [189-193]. Although the link between acidosis and RANK/NFATc1 mediated carcinogenesis or tumor promotion is not established, chronic activation of these factors through a dietary induced state of dysregulated acid-base status may contribute to cancer risk.

## Conclusion

This work examines the potential for cancer risk or tumor promoting consequences of diet-induced acidosis. Although protein is a major factor involved in promoting endogenous acid production, it should be made clear that attenuation of protein consumption is not a recommended dietary strategy for attaining improved acid-base balance. There is scientific evidence supporting the concept that appropriate alkali supplementation in the form of fruits and vegetables serves aptly to neutralize excess [H^+^ produced from protein metabolism [34,194]. The analysis provided discusses how diet-induced acidosis is a potential upstream and indirect trigger in a multifactorial cascade of molecular events associated with carcinogenesis. There is limited evidence to suggest that dietary acidosis alone is sufficient in increasing cancer risk, but it may function in concert with other factors associated with cancer risk. Obesity or metabolic syndrome, which effect glucocorticoid and adipokine profiles and are often linked to insulin resistance and the pro-inflammatory state, could also serve as significant factors as they are associated with both acidogenic or ‘Western’ diet [34] and cancer risk [3].

In conclusion, there are numerous systemic pathways affected by diet-induced acidosis that may be cancer promoting, but a causal role is poorly defined. Moreover, the contribution of diet-induced acidosis in driving carcinogenesis would be difficult to measure especially since the effects appear to accumulate for a long period of time. Nonetheless, exploring the role of dietary induced acidosis involvement in molecular pathways that promote carcinogenesis will raise new questions and foster ideas to improve our understanding on the role of acid-base balance in human disease.

## Abbreviations

IGF-1: Insulin growth factor; GLUT: Glucose transporter; IDO: Indoleamine 2,3-dioxygenase; TDO: 2,3-dioxygenase; APC: Antigen presenting cell; AHR: Aryl hydrocarbon receptor; ROS: Reactive oxygen species; CKD: Chronic kidney disease; BMI: Body mass index; PDGF-BB: Platelet derived growth factor-BB; OGR1: Ovarian cancer G-protein coupled proton-sensing receptor; NFATc1: Nuclear factor of activated T-cells; AMP-k: Adenosine monophosphate-activated protein kinase; PPARa: Peroxisome proliferator-activated receptor alpha; MEP: Mitochondrial energy production; MAPK: Mitogen-activated protein kinase; 11ßHSD2: 11ß-hydroxysteroid dehydrogenase 2; HB EGF: Heparin-binding epidermal growth factor-like growth factor; RANKL: Receptor activator of NFкB ligand; OGR1: Ovarian cancer G-protein coupled proton-sensing receptor; NFATc1: Nuclear factor of activated T-cells; AMP-k: Adenosine monophosphate-activated protein kinase; PPARa: Peroxisome proliferator-activated receptor alpha; MEP: Mitochondrial energy production; MAPK: Mitogen-activated protein kinase; 11ßHSD2: 11ß-hydroxysteroid dehydrogenase 2; AICR: American Institute for Cancer Research.

## Competing interests

The author declares that he has no competing interests.

## Authors’ contributions

IR conceived and drafted the manuscript in its entirety and approves the final manuscript.
